# Artificial neural network based forecasting of diesel engine performance and emissions utilizing waste cooking biodiesel

**DOI:** 10.1038/s41598-024-71675-x

**Published:** 2024-09-20

**Authors:** M. S. Gad, H. E. Fawaz

**Affiliations:** 1 Mechanical Engineering Department, Faculty of Engineering, Fayoum University, Fayoum, Egypt; 2 Mechanical Engineering Department, National Research Centre, Giza, Egypt

**Keywords:** WCO biodiesel, Performance, Emissions, Artificial neural networks, Multi-layer perceptron, Back-propagation, Engineering, Mechanical engineering

## Abstract

Ecological and environmental problems resulting from fossil fuels are due to the harmful emissions released into the atmosphere. The rising interest in searching for alternative fuels like biodiesel is growing to solve these problems. Waste cooking oil (WCO) is transformed into methyl ester and combined with biodiesel in percentages of 25, 50, 75, and 100%. Research is done on the impacts of methyl ester blends on engine performance and emissions. Compared to diesel, the methyl ester combination showed 25% lower brake power and 24% loss in thermal efficiency at maximum load and 1500 rpm. However, diesel fuel showed 23% lower specific fuel consumption increase than biodiesel. Compared to diesel, methyl ester exhibits 15% lower air-fuel ratio and 4% volumetric efficiency. Biodiesel lowers CO, HC, and smoke concentrations by 12, 44, and 48%, respectively, compared to diesel. Biodiesel emits 23% higher NOx at 1500 rpm and 100% engine load. To predict the emissions and performance of different percentages of biodiesel at engine speed variation, an artificial neural network (ANN) model is presented. ANN modeling minimizes labor, time, and finances and uses nonlinear data. Predictions were produced about the brake output power, specific fuel consumption, thermal efficiency, air-fuel ratio, volumetric efficiency, and emissions of smoke, CO, HC, and NOx as a function of engine speed and blend ratio. All correlation coefficients (r) over 0.99 and $R^{2}$ values were beyond 0.98 for all variables. There were low values of MSE, MAPE, and MSLE with significant predictive ability. WCO’s biodiesel is a viable diesel engine replacement fuel.

## Introduction

Finding alternative diesel engine fuels is becoming increasingly important due to environmental concerns and the depletion of fossil resources. Biodiesel is one solution to these problems. Methyl ester is characterized by its renewable nature, biodegradability, non-toxicity, and absence of sulfur and aromatics^1–3^. Because vegetable oils have higher density, viscosity, and reduced volatility, they are not recommended for direct use in diesel engines. Its properties resulted in inadequate spray penetration and fuel atomization. Problems with spray penetration were caused by thickening lubricating fluid, piston ring sticking, and engine deposits^2–4^. The properties of oil can be enhanced via a process known as transesterification. Unlike biodiesel, diesel fuel produces higher pollutants^5–7^. The temperature dropped as the additive volume % increased, affecting the fuel’s heat dispersion and thermal conductivity^8,9^. Several researchers who studied the blends discovered that the peak cylinder pressure inside the combustion chamber dropped as the percentage of biodiesel in the mixture increased^10,11^. Blends that had higher percentage of biodiesel released heat at slower rate during the premixed combustion stage. Pure forms of vegetable oils such as Neem seed oil, fish oil, palm, pogoamia, jatropha, and others were used^12–14^.

By utilizing two ANN training procedures to compare the predicted and experimental results, robustness and adaptability were examined^15,16^. Non-linear mapping was used to enhance multi-layer perception. For exhaust gas temperature, specific fuel consumption, thermal efficiency, HC, NO_X_, and smoke emissions, there were correlation values of 0.995, 0.980, 0.999, 0.985, and 0.999, respectively^17^. With a correlation value between 0.97 and 0.99, engine performance and emissions were calculated using the ANN model closely match experimental data^18^. The engine performance and emissions are predicted using a feed-forward back propagation artificial neural network model based on palm biodiesel. R > 0.99 and a mean absolute error of 1.879% were employed in the ANN model^19,20^. The ANN model, which is based on the back-propagation learning technique, is used to predict the engine parameters. The corresponding R values for training, testing, and validation were 0.9994, 0.9995, and 0.9999, respectively. Forward-Looking Backward Propagation Utilized is the Levenberg-Marquardt ANN model method, which has three layers and MSE of ten neurons^21^. In ANN modeling, higher correlation coefficient values (R^2^) were observed, ranging from 0.88 to 0.95. There was also minimal root mean square error (RMSE) and mean relative error (MRE). For engine performance and emissions prediction and optimization, the ANN model produced the best results^22,23^. The performance and combustion outputs were 0.3 kg/kWh specific fuel consumption (BSFC), 30.8% thermal efficiency (BTE), and 65.6 bar peak cylinder pressure at these optimal operating parameters. There were 26 ppm of HC, 853 ppm of NOx, and 0.0107% of CO emissions. Every outcome was within 7 and 7% of what the model predicted^24^. An extreme learning system based on kernels predicts engine performance and emissions. The average absolute percentage errors for BSFC, BTE, CO, NOx, and smoke opacity were 1.363, 1.482, 4.597, 2.224, and 2.090%, correspondingly^25^. The response Surface technique was created statistically using Central Composite Design. The load should be 14.49 N.m., and the methyl ester ratio of JP-8 fuel should be 2.47%. In the output parameters, the BSFC was 193.46 g/kWh, and the CO, IMEP, COVimep, and NOx values were 724 ppm, 3.71 bar, and 168.62 ppm, respectively^26^. Using the obtained data sets, the ANN model is tested and trained, and its performance is evaluated using R^2^ and RMSE. The R^2^ range is almost unity, indicating that the proposed network can accurately predict the required combustion parameters^27^. Nonlinear regression with a reasonably modest root mean square error and a correlation coefficient (R) between 0.95 and 0.99 can be used to predict the emission characteristics and performance. The optimization procedure operates in the mixture range of 0 to 20%. The optimal mixture is 13% biodiesel to diesel with an injection time of 240 BTDC where performance and emissions are given equal weight^28^.

Radial-basis function neural network (RBFNN), the extreme learning machine (ELM), and the least-squares support vector machine (LS-SVM) were the three neural networks that were employed. In the presence of the logarithmic transformation, LS-SVM and RBFNN perform worse than ELM. PSO performs better than SA when computing times are reasonable^29^. An extreme Learning Machine with Wavelet Transform Algorithm (ELM-WT) was created to forecast engine performance. The approach correctly predicted the engine performance with a root-mean-square error of less than 2.79, Pearson correlation value of greater than 0.97, and coefficient of determination better than 0.95^30,31^. The engine under test ran more smoothly and produced less pollutants when the engine blend of 20.22%, the engine speed of 1483.39 rpm, and the engine load of 95.6% were applied. Values of BTE, BSFC, BSEC, CO, HC, and NOx were, respectively, 20.61%, 0.32 kg/kWh, 6.14 MJ/kW, 0.08963%, 18.28 ppm, and 347.72 ppm^18,32^. Using an ANN model, diesel engine performance and emissions were predicted for engine load, biodiesel percentage, and concentration of ZnO nano additive. The fifteen hidden neurons converged in 0.00452 s, with a mean square error (MSE) of 0.00128^33,34^. Performance and emissions of diesel engines were predicted using the ANN model for engine load, methyl ester percentage, and nanomaterial concentration. For BTE and BSFC, the coefficients of determination were over 98% and over 99%, respectively. The engine produces much less CO, HC, and smoke (8.26, 2.08, and 3.08%, respectively) when it operates on Sterculia foetida biodiesel SFB5. Conversely, there were rises of 22.39% and 3.53% in NO_X_ and CO_2_ respectively. Extreme learning machine modeling performs better than artificial neural networks in forecasting engine emissions and performance^35,36^. The optimal emissions parameters and engine performance have been calculated using the Taguchi technique. Multiple regression and ANN have been used to evaluate the error. For thermal efficiency, NOx, hydrocarbons, and CO_2_, the percentage errors ranged by 4.6%, 1.26%, 2.96%, and 29.05%, respectively; the errors were highest for BSFC and CO. The ideal regression model has an acceptable accuracy of 18.482, an adjusted R^2^ of 0.972, and a standard deviation of 0.095. With values ranging from 0.9947 to 0.9997, the performance and emission measures showed significant correlation, and the Kling-Gupta efficiency was higher than 98%. The BRT model is superior to ANN-model^37^.

Exhaust Gas Recirculation (EGR) system, diesel, biodiesel, hydrogen, and aluminum oxide nanoparticles (Al_2_O_3_) were proven to have an impact on diesel engine emissions and performance. Carbon monoxide (CO) and hydrocarbons (HC) fell by 14.9% and 11.7%, respectively, with 30% biodiesel. Al_2_O_3_ concentration increased from 30 ppm to 60 ppm, whereas HC content was dropped by 5.4%. Nitrogen oxides (NOx) were increased by 8% but CO was reduced by 5.8% with a further increase to 90 ppm. But there was 4.89% gain in torque. Power was enhanced by 16% when hydrogen was added to the intake air volume, but CO was also raised by 7.19%^38^ The literature review indicates that previous studies mostly examined the influences of methyl ester/diesel mixtures on diesel engine performance and emissions. Since disposal of waste cooking oil (WCO) is more expensive, contaminates water sources, and hurts the environment, this endeavor employs non-edible oil that produces more biodiesel. Non-edible oil with higher methyl ester output is used instead of waste cooking oil. This waste oil may be transformed into renewable sustainable useful energy. This alternative fuel supports ecologically friendly energy sources and satisfies the Sustainable Development Goals (SDGs). WCO was converted into biodiesel using the transesterification process, and various volume ratios of 25, 50, 75, and 100% were then added to diesel oil. ASTM showed that the properties of the methyl ester mixtures are comparable to those of diesel oil. According to the research, there is a gap in the literature that is not addressed. An artificial neural network (ANN) was developed using a new dataset based on the present trial data to predict engine emissions and the performance of various blends of WCO biodiesel and pure diesel at varying engine speeds.

The feasibility and accuracy of the ANN model in predicting biodiesel performance and emissions were investigated. This reduces the reliance on physical testing, associated challenges, and redundancies. An accurate representation of the complex interactions inside the engine system is made possible by the great efficacy of ANNs in capturing complicated linkages. Diesel engines become more responsive and adaptable when ANN models are implemented in real-time and allow the engine to change its operating circumstances dynamically. The goal is to create an ANN model that minimizes labor, time, and financial losses for this reason. The work’s primary goal is to fulfill the aforementioned qualities that are absent from the literature. ANN is the preferred way since it handles complex, noisy, and non-linear data regarding other approaches. It requires less formal statistical training. We use this strategy since the experimental data is nonlinear. Neural networks analyze information faster than conventional computers and are more adept at seeing patterns and solving problems. Therefore, less testing is required to establish if biodiesel is suitable for use in diesel engines. By contrasting the results of the modeling techniques with the trial outcomes, the prediction model’s accuracy was demonstrated. By contrasting the results of the modeling techniques with the trial outcomes, the prediction model’s accuracy was demonstrated. Among the engine performance parameters that were evaluated were fuel-air equivalency ratio, brake power, specific fuel consumption, thermal efficiency, and volumetric efficiency. Research on exhaust emissions, such as smoke, CO, HC, and NOx, has been done. Because it can forecast engine characteristics under different circumstances, the provided artificial neural network model is quite significant.

## Methodology

### Biodiesel production

Waste cooking oil (WCO) is utilized directly in CI engines because of its high viscosity. The methyl ester and the oil were separated during transesterification. WCO was filtered, and the oil was heated to 110 degrees Celsius to remove moisture. Next, the oil was poured into a flask supported by a thermometer, condenser, and magnetic stirrer. Methoxide is created by dissolving 1.5% by weight of potassium hydroxide (KOH) in 1:9 molar mixture of methanol and alcohol. The oil and methoxide mixture were aggressively stirred for 90 min at $60$ °C to create the methyl ester and glycerin. To extract the ester and glycerin, the blend was kept in the separating funnel for an entire day. With warm water, the catalyst, contaminants, and unreacted methanol were removed. The biodiesel was dried using a rotary evaporator once the water had been removed. The volumetric ratios of diesel oil and methyl ester are 25, 50, 75, and 100%. The features of the biodiesel blends are shown in Table 1 alongside those of crude diesel.

**Table 1:** WCO biodiesel and its mixtures properties.

Properties	Method	Biodiesel WCO	Diesel
Density $@15^{\circ }C$	ASTM D-4052	883	835
Kinematic viscosity, cSt, $@40^{\circ }C$	ASTM D-445	5.1	3.5
Flashpoint, °C	ASTM D-93	120	72
Cetane number	ASTM D-13	52	49
Lower calorific value, MJ/kg	ASTM D-224	39.5	42

### Experimental test rig

**Fig. 1:**
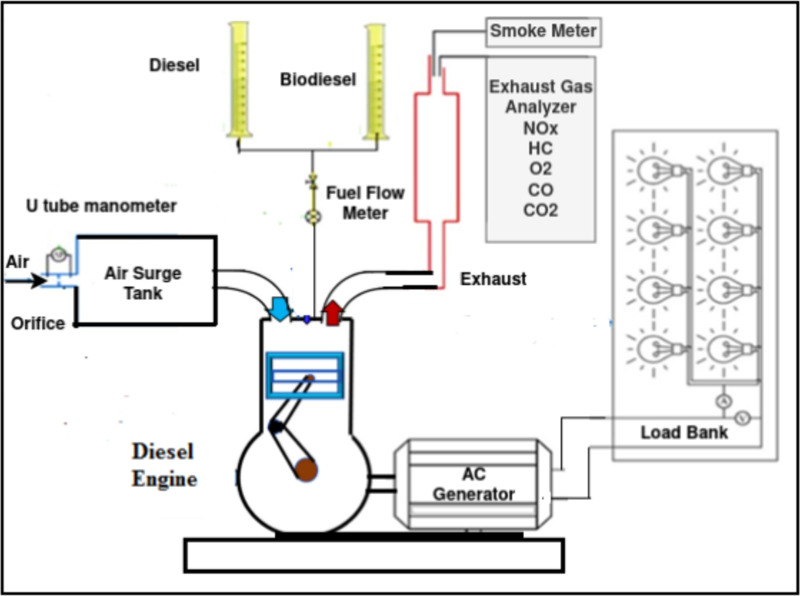
Schematic diagram of experimental setup.

The engine under evaluation is a single-cylinder DEUTZ F1L511 type, including 105 mm stroke length, 100 mm bore, 17.5% compression ratio, 240 BTDC fuel injection advance angle, and 5.775 kW rated output at 1500 rpm. Figure 1 shows the schematic diagram of the used experimental setup. The test engine was connected to an AC generator (Model: Meccalte, U.K.) with the highest output power of 10.5 kW to show the load of the engine. An orifice with a sharp edge that is mounted on the air box measures the intake air flow rate. The fuel flow rate was calculated as the time required to consume a fixed, measured fuel volume of 25 cm^3^. Calibrated K-type thermocouples were utilized to measure the supply air and exhaust gas temperature. The German-made MRU DELTA 1600-V gas analyzer was used to monitor emissions of CO, NOx, and HC. Measurements of smoke were made with a smoke meter (Model: OPA, France).

Cylinder pressure up to 250 bar was measured using a water-cooled Kistler piezoelectric pressure transducer (model 601A), which had a sensitivity of 16.5 pc/bar and an accuracy of 1.118 % (2692-A-0S4). The piezoelectric pressure transducer was flush placed with the cylinder head to eliminate pressure signal lag and prevent pipe connecting passage resonance. To determine the piston position, a proximity switch (Type LM12-3004NA) was positioned on the engine’s output shaft was employed. Over 120 engine cycles, the pressure values were averaged. The pressure crank angle diagrams were captured and analyzed to produce the combustion parameters using a data acquisition system. The high-speed data was collected using LABVIEW software and a National Instruments data acquisition card for later processing (NI-USB-6210).

The engine utilized for the tests was fully loaded and runs between 800 and 1800 rpm. The engine was then warmed up using only pure diesel fuel, without being filled up, until the exhaust temperature stays constant. The engine load was controlled once the fuel line that was tested is switched. After the condition of affairs becomes stable, readings are ultimately taken. Every test consists of three measurements taken to ensure that repeatability errors are detected. Specific operational aspects and procedures, such as equipment selection, climatic conditions, and instrument calibration, are the source of errors and uncertainties in the experiments. To measure the overall correctness of the experimental inquiry, error analysis was employed. Every test was run thrice to guarantee the accuracy and consistency of the findings. The thermocouples’ noteworthy range included 0 to 1300 K, with an accuracy of ± $1$ °C and an uncertainty of ± 0.15. The brake power indicator had an accuracy level of ± 10 W and an uncertainty of ± 0.2, covering a range of 250 to 7000 W. The ranges for exhaust gas measurements were as follows: CO (0–10%), HC (0–20,000 ppm), and NO (1–5000 ppm). For each gas, there was an uncertainty of ± 1. Based on the calculation of Equation 1, the total uncertainty of the experiment was found to be a maximum of 3.33%.

1 $$\begin{aligned} W_{R}= \sqrt{{\bigg (\frac{\partial {R}}{\partial {X_1}}\delta {X_1}\bigg )}^2 + {\bigg (\frac{\partial {R}}{\partial {X_2}}\delta {X_2}\bigg )}^2 +\cdots + {\bigg (\frac{\partial {R}}{\partial {X_n}}\delta {X_n}\bigg )}^2} \end{aligned}$$

The whole uncertainty of the test rig is represented by $W_R$. The independent and dependent measurements are indicated by the parameters R and X, respectively.

### Artificial neural network methodology

**Fig. 2:**
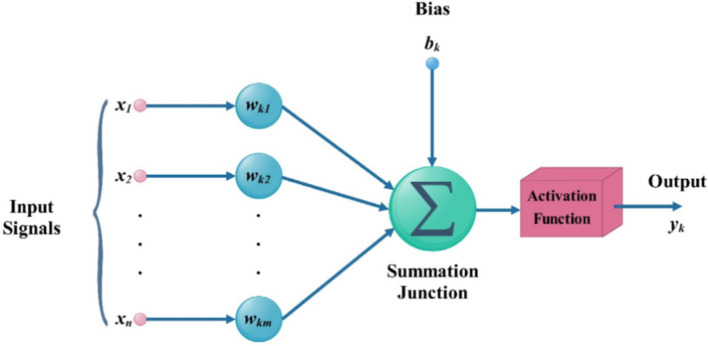
ANN configuration.

Given a collection of experimental input-output data, it is useful to develop a mathematical model that can be used to predict engine performance and exhaust emissions for different combinations of engine speed and fuel mixtures. This saves the time and effort which would be required to repeat the experiments. In this study, engine performance and emissions parameters are predicted as a function of engine speed and biodiesel ratio using an artificial neural network (ANN). ANNs are mathematical representations of neuronal behavior in biological systems, especially in the brain^39,40^. An ANN’s unit element is a single neuron, which is schematically shown in Fig. 2.

The motivation for using Artificial Neural Networks (ANNs) over other modeling techniques for regression problems in predicting diesel engine performance and emissions is driven by their superior ability to handle the complex, nonlinear interactions inherent in engine dynamics and emission processes. Diesel engine performance and emissions are influenced by a multitude of interdependent factors that exhibit intricate nonlinear relationships that traditional regression methods often fail to capture adequately. ANNs excel in this context by automatically learning and extracting relevant features from the data, reducing the need for extensive domain-specific feature engineering. Their flexibility allows for the integration of various types of input data, including time series and sensor data, which are common in engine monitoring systems. Additionally, ANNs’ robust generalization capabilities ensure accurate predictions across different operating conditions, enhancing their reliability in real-world applications. With the support of advanced computational resources and neural network frameworks, ANNs can be efficiently trained on datasets, accommodating the high dimensionality and noise typically present in engine performance and emissions data. This makes ANNs an ideal choice for developing predictive models that can optimize engine performance while minimizing emissions, aligning with stringent environmental regulations and efficiency goals.

Numerous ANN types with various design patterns and ranges such as feed-forward, recurrent, feedback, classification, radial basis functional, sequence to sequence models, modular, and other ANNs types, are shown by the literature review^41–44^. This paper uses the feed-forward ANN. An ANN of this type can perform several stages of work for a single hidden neuron:

i. The neuron receives the input parameters $x_i$ together with the appropriate weight coefficients, or signal value, $w_i$. It is necessary to introduce the weight coefficient values during the initial estimation.
ii. The sum *S* is the result of a linear addition of the bias *b* and the signal values. The magnitude of the bias determines the minimum value of *S* at which the neuron reacts to incoming information.
  2 $$\begin{aligned} S = \sum _{i = 1}^{m} {{x}_{i} {w}_{i}} + b \end{aligned}$$
  The computed value of *S* is going through activation function $\Phi$ and being sent to subsequent neurons, or it is represented by predicted value $\widehat{{y}_{i}}$ of a known variable ${y}_{i}$. In the context of this work, fully linked feed-forward ANNs are considered. This suggests that every neuron in the preceding layer sent a signal to every other neuron in the following layer. A typical ANN consists of $\zeta$ total layers ($\zeta -2$ hidden layers), with $n^\zeta$ neurons in each layer. The number of neurons in the previous layer($\zeta -1$) will be assigned index *i*, while the current layer ($\zeta ^th$) will be assigned index *j*. The signal magnitude $S_{ij}^\zeta$ at a certain neuron *j* at the $\zeta ^th$ layer is determined as:
  3 $$\begin{aligned} S_{ij}^\zeta =\Phi \left( \sum _{i = 1}^{n^{\zeta -1}}{x_{i}w_{ij}+b_j}\right) \end{aligned}$$
  Where $n^\zeta$ is the neuron’s number at $\zeta ^th$ layer. Rectified linear unit activation function^45^ was utilized for the hidden layers as:
  4 $$\begin{aligned} \Phi \left( S\right) =max\left( 0,X\right) \end{aligned}$$
  while at the output layer, the linear function was applied as:
  5 $$\begin{aligned} \Phi \left( S\right) =X \end{aligned}$$
  The predicted vector of output parameter $\widehat{Y}$ is computed by calculating Eq. (3) across all ANN layers. The ANN training procedure aims to obtain the proper weights $w_{ij}^\zeta$ and biases $b_{j}^\zeta$ that can get the minimal loss function, or distance *E*, between the sets of the actual variable values *Y* (experimental data) and its corresponding predicted values, $\widehat{Y}$. The mean squared error (*MSE*) serves as the loss function in the present model. Numerous techniques can be used for loss functions minimization, including Nesterov momentum modification, adaptive subgradient approach, stochastic gradient descent and adaptive moment estimation algorithm(*ADAM*)^40–46^. The *ADAM* technique which accelerates the *ANN* training was used in the present model. The values of input parameters can fall within various ranges. For instance, the maximum blend ratio is 100% while the maximum engine speed is 1800 rpm. This problem causes stark differences in the loss function gradients regarding weights value $\frac{\partial E}{\partial w_{ij}^\zeta }$ which causes a relatively large training time increase. The prescribed problem was solved in the current model using the “$Min-Max$” normalization algorithm^47,48^. So, all of the input data was first normalized using the following formula:
  6 $$\begin{aligned} X_{norm}=\frac{X-min(X)}{max(X)-min(X)} \end{aligned}$$
  Consequently, every input variable has a range from 0 to 1 $\left( X_{norm}=(0,1)\right)$. Total number of impacted variables *I* could be obtained by adding the weights $w_i$ and biases $b_i$ which are related to the number of neurons $n^\zeta$ at $\zeta ^{th}$ layer as:
  7 $$\begin{aligned} I={\left( n^\zeta \right) }^2+\sum _{i = 2}^{\zeta -1} {n^{i}.n^{i+1}}+\sum _{j = 1}^{\zeta -1} {n^{j}+n^{\zeta -1}.n^\zeta } \end{aligned}$$
  The ANN prediction accuracy is observed to be affected by more parameters when more neurons are used. However, this increase in neuron number could result in the overfitting issue^41^. Training and validation datasets are often separated apart from the broader dataset. Following that, for both the training and validation datasets, the loss function is minimized. Such an approach diminishes the risk of overfitting when the data over over-predicted by ANN^41^. In the context of this work, the proportion of training and validation datasets was 20% and 80% of a general dataset, respectively.

### Training the artificial neural network

In the present study, a dataset comprising 25 data points, uniformly distributed across the input feature space was utilized, for training an Artificial Neural Network (ANN) for predicting diesel engine performance and emission characteristics. Each input feature was sampled from a uniform distribution, ensuring equal representation across the entire range of possible values. This uniform distribution was chosen to provide a balanced and comprehensive coverage of the input space, which aids in mitigating any potential bias and improving the generalization capability of the ANN. The dataset was normalized to enhance the training efficiency and model performance. Despite the relatively small size of the dataset, the uniform distribution and normalization ensured that the ANN could learn the underlying patterns effectively, facilitating accurate and robust predictions of diesel engine performance.

**Fig. 3:**
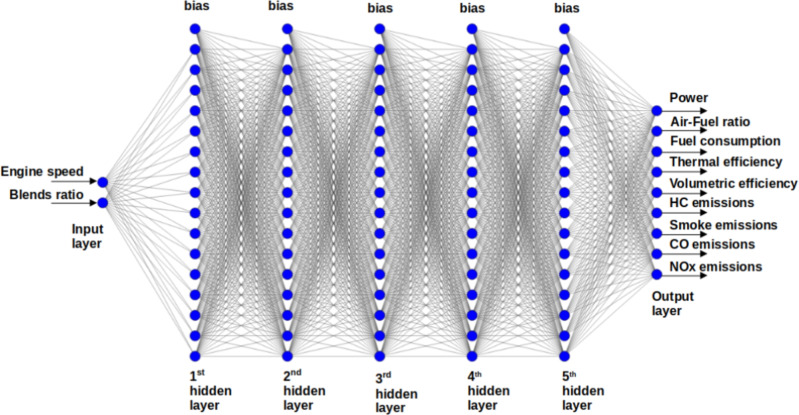
Schematic diagram of the neural network architecture.

**Fig. 4:**
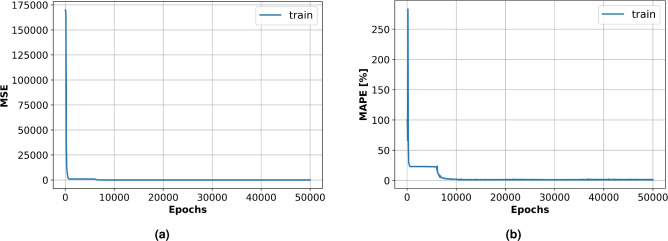
A convergence of the ANN training (**a**) MSE and (**b**) MAPE.

Forward propagation of the input variables, loss function estimation, and backpropagation technique modification of the biases and weights comprise the training process. Such a procedure was repeated for 50,000 epochs to ensure the loss function convergence. The program code was written in Python 3.0 using a Tensor Flow ecosystem with the open-source ANN library Keras^49^. In the current model, a multi-layer perceptron which has several layers with different amounts of neurons on each one has been used^50–54^. ANN training process should begin with the initialization of training variables, such as weights and biases. In the current model, the truncated normal distribution approach was used for this. Ascertaining the total number of neurons required for loss function minimization (the number of influencing parameters) was carried out through a series of comparisons between the training and validation datasets’ loss function values. In the present model, the input layer is composed of two neurons that represent Engine speed and blend ratio. Following the input layer, five hidden layers each one consisting of 16 neurons were employed, while the output layer contains nine neurons. These neurons represent the engine performance and emission characteristics such as brake output power, brake-specific fuel consumption, brake thermal efficiency, volumetric efficiency, air-fuel ratio, CO emission, HC emission, NOx emission, and smoke emission. Thus, the ANN structure follows as 2-16-16-16-16-16-9 format as shown in Fig. 3. Consequently, *I* = 1289 parameters were employed, which provided the MSE and MAPE of 5.0943 and 1.1035% respectively as displayed in the convergence graphs shown in Fig. 4.

In this study, the architecture of the Artificial Neural Network (ANN) was optimized through an iterative process of successive comparison of the loss function values on the training datasets^55^. After evaluating various configurations, an optimal structure consisting of 5 hidden layers, each with 16 neurons, was identified. This determination was based on a systematic approach that involved adjusting the number of hidden layers and neurons and comparing the resulting performance metrics. The selected architecture provided the best balance between model complexity and predictive accuracy, as evidenced by the minimized loss function values during the training process.

### Performance accuracy

Mean Squared Error (*MSE*), Mean Absolute Percentage Error (*MAPE*), Mean Squared Logarithmic Error (*MSLE*), Coefficient of Determination $(R^2)$, and Correlation Coefficient (*r*) were the five metrics utilized to assess the model’s prediction accuracy. These indicators offer several viewpoints on the models’ performance. Low values of *MSE*, *MAPE*, and *MSLE* demonstrate high model accuracy. High scores of Coefficient of Determination $R^2$ and Correlation Coefficient *r* near to “one” denote a robust relationship between the experimental and predicted values, with “one” denoting an ideal prediction. While it is challenging to get perfect zeros of the error metrics(*MSE*, *MAPE*, and *MSLE*), models with nearly zero values, as well as higher $R^2$ and *r* scores, can be considered superior.

Mean Squared Error (*MSE*) is the average of the squares of the deviations between the experimental and model prediction values (Eq. 8)

8 $$\begin{aligned} MSE = \frac{1}{N} \sum _{i = 1}^{N} {{\left( {y}_{i} - \widehat{{y}_{i}} \right) }^{2}} \end{aligned}$$

where *N* is the number of data points.

The error percentage number is calculated using the Mean Absolute Percentage Error (*MAPE*), which is the mean of the absolute discrepancies between the experimental data and the model predictions divided by the experimental data and multiplied by “100” (Eq. 9).

9 $$\begin{aligned} MAPE = \frac{1}{N} \sum _{i = 1}^{N} \frac{|{y}_{i}-\widehat{{y}_{i}}|}{ |{y}_{i}|} * 100 \end{aligned}$$

Mean Squared Logarithmic Error (*MSLE*) is the relative deviation between the experimental and predicted log-transformed values. (*MSLE*) attempts to handle minor deviations between tiny experimental and predicted values like that of considerable deviations between big experimental and predicted values (Eq. 10).

10 $$\begin{aligned} MSLE = \frac{1}{N} \sum _{i = 1}^{N} {{\left( log \left( {y}_{i} + 1 \right) - log \left( \widehat{{y}_{i}} + 1 \right) \right) } ^ {2}} \end{aligned}$$

The proportion of variance in a predicted value that can be estimated by experimental data is known as the Coefficient of Determination $(R^2)$, which is a statistical measure that indicates the degree to which the regression model, can predict the dependent variable, or the results of observed data (Eq. 11).

11 $$\begin{aligned} R^{2} = \frac{SSR}{SST} = \frac{\sum _{i = 1}^{N} {{\left( \widehat{{y}_{i}} - {\bar{y}} \right) } ^ {2}}}{\sum _{i = 1}^{N} {{\left( {y}_{i} - {\bar{y}} \right) }^{2}}} \end{aligned}$$

Estimating the Correlation Coefficient (*r*) offers a straightforward equation that is readily applicable to the ANN model predictions $(\widehat{y_i})$ and its corresponding experimental values $(y_i)$ (Eq. 12).

12 $$\begin{aligned} r = \frac{\sum _{i = 1}^{N}{\left( {y}_{i}-{\bar{y}} \right) }{\left( \widehat{{y}_{i}}-\bar{\widehat{y}} \right) }}{ \sqrt{ \sum _{i = 1}^{N}{\left( {y}_{i}-{\bar{y}} \right) }^2 \sum _{i = 1}^{N}{\left( \widehat{{y}_{i}}-\bar{\widehat{y}} \right) }^2 } } \end{aligned}$$

All of the previously listed metrics provide important information on the accuracy, consistency, and reliability of the models, which makes an all-encompassing evaluation of their effectiveness possible.

## Results and discussion

### Brake output power

**Fig. 5:**
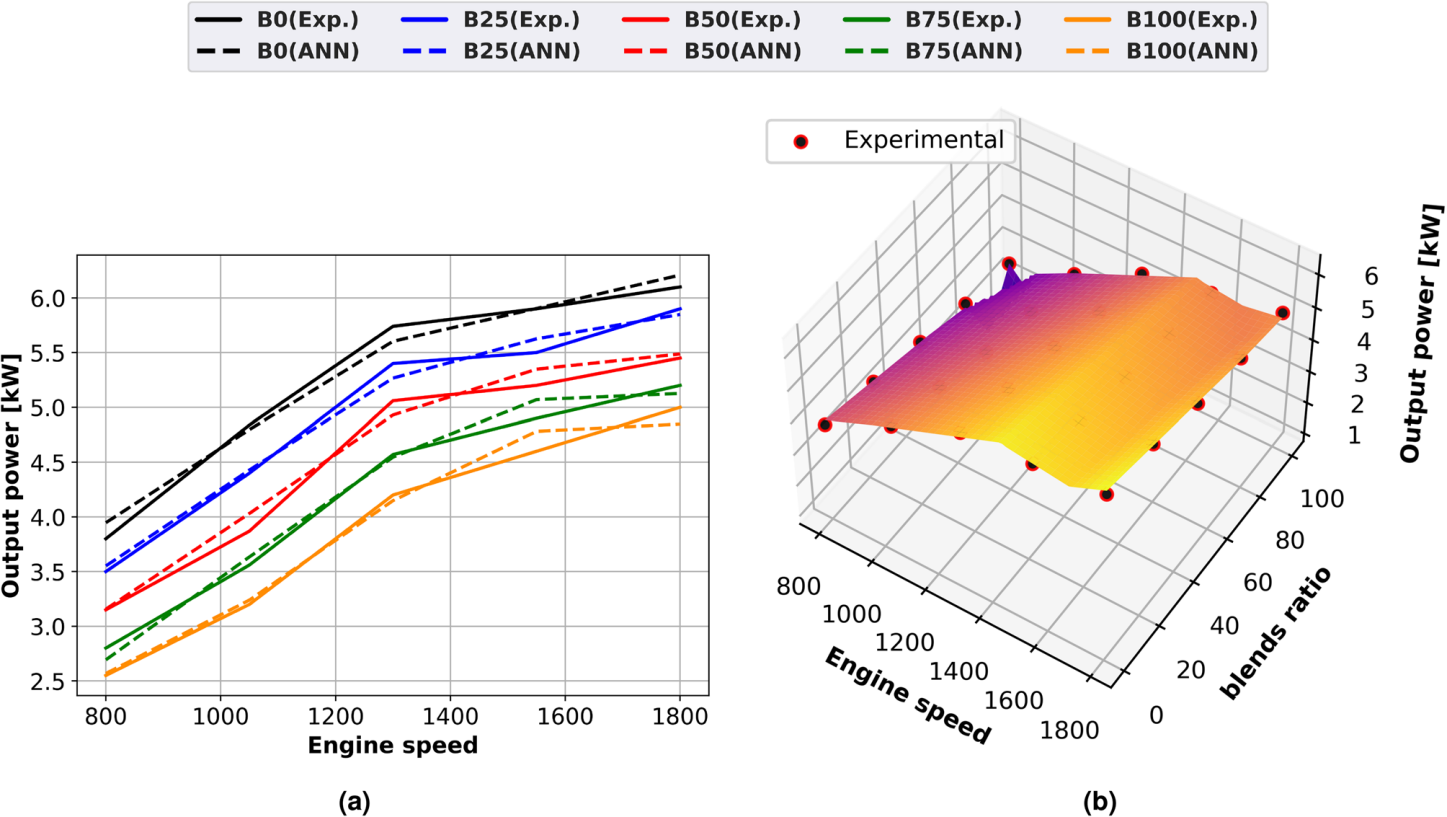
Output power of methyl ester blends at different engine speeds (**a**) line plots for Experimental and ANN predicted, (**b**) performance, (**c**) experimental scatter and surface plots of the ANN model.

Figure 5 shows how biodiesel mixtures affect engine brake power for both experimental and model prediction using surface plots with experimental scatter (Fig. 5b) and line graphs (Fig. 5a). Engine speed and brake power increase together. The amount of fuel used increases as engine speed climbs. As the amount of biodiesel grows, the output power drops because methyl ester has a lower calorific value about diesel oil. Because of its increased density and viscosity, biodiesel can cause problems with fuel atomization, vaporization, and fuel-air mixing compared to pure diesel. Diesel fuel mixtures including biodiesel require more fuel to produce the same output power. The lowest output power of 100% methyl ester (B100) is 25% less than that of diesel oil at full load and 1500 rpm.

### Brake specific fuel consumption (BSFC)

**Fig. 6:**
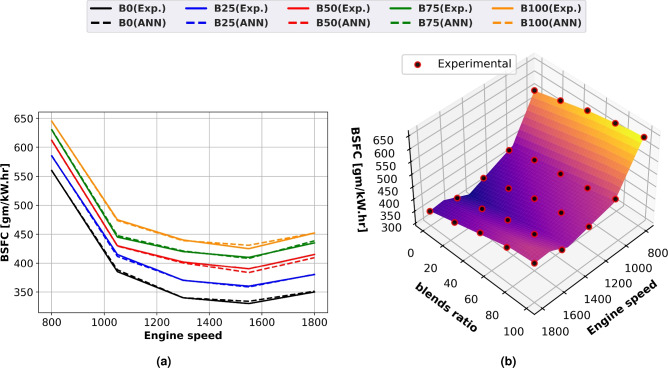
BSFC of biodiesel mixtures at engine speed variation (**a**) line plots for Experimental and ANN predicted performance, (**b**) experimental scatter and surface plots of the ANN model.

Figure 6 illustrates the change in BSFC with engine speeds for diesel and biodiesel blends for experimental and predicted findings using line graphs (Fig. 6a) and surface plots with experimental (Fig. 6b). For all loads, diesel has lower specific fuel consumption than biodiesel. With biodiesel, the engine has to use more fuel to produce the same amount of power since it has a lower calorific value than diesel fuel. The primary causes of biodiesel’s increased specific fuel consumption are its low volatility, poor combustion, and higher viscosity. Due to atomization problems, heterogeneous mixing, and incomplete combustion caused by molecular friction, BSFC values were increased in diesel oil. At an engine speed of 1500 rpm, biodiesel showed the largest BSFC gain of 23% over diesel oil.

### Brake thermal efficiency (BTE)

**Fig. 7:**
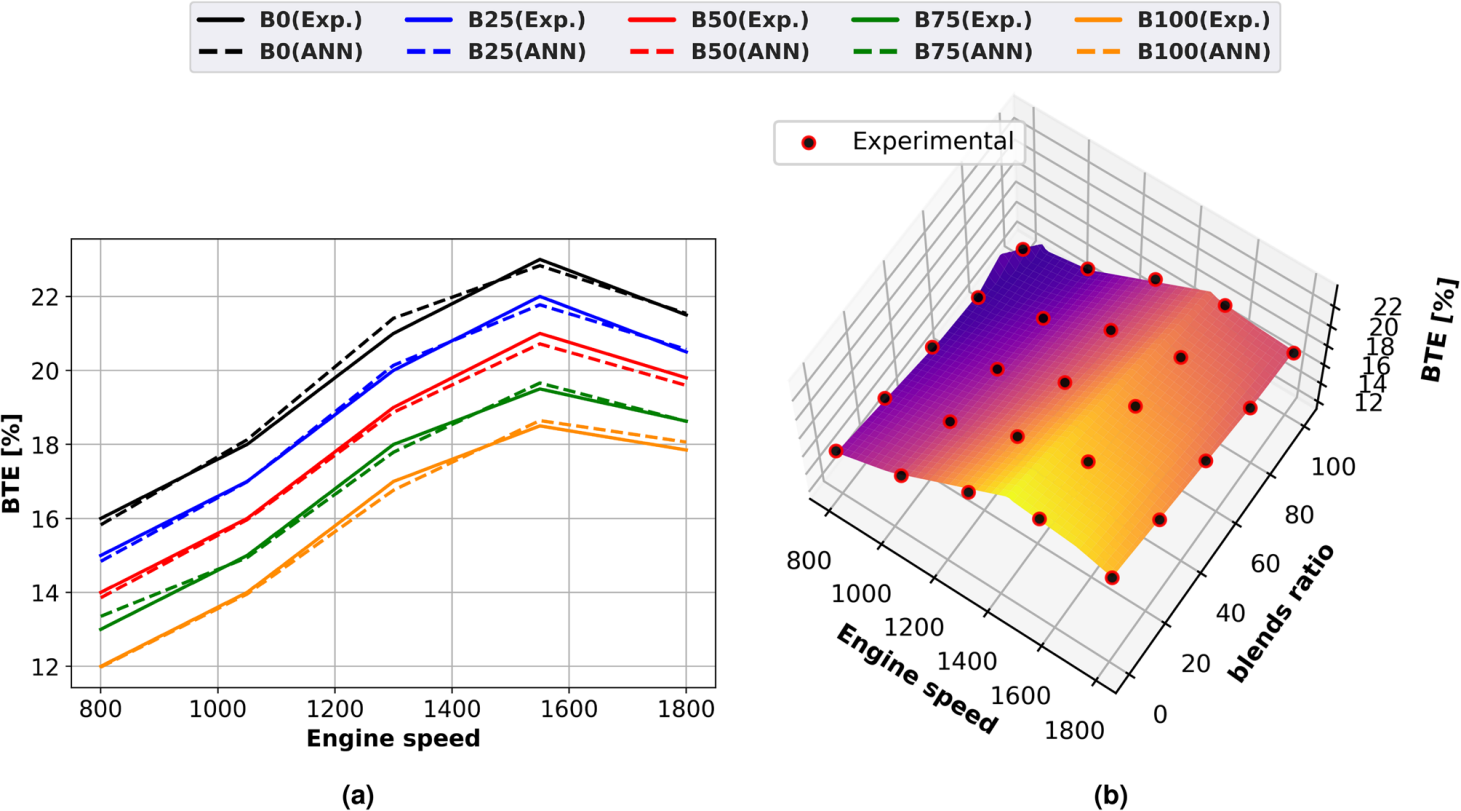
Thermal efficiency of biodiesel mixtures at different engine speeds (**a**) line plots for Experimental and ANN, predicted performance, (**b**) experimental scatter and surface plots of the ANN model.

Using line plots and surface plots with experimental scatter, Fig. 7 illustrates the decrease in BTE for both experimental and expected findings for diesel and biodiesel blends, as shown in Fig. 7a and b, respectively. As engine speed increases, so does fuel consumption and engine power. As the methyl ester ratio rises, the thermal efficiency falls. BTE peaks as engine load rises and then starts to fall. Reduced engine speeds result in increased heat loss and concentrated fuel use. Because of increasing fuel consumption and friction losses, specific fuel consumption increases with engine speed. Biodiesel has a lower thermal efficiency due to its increased viscosity, weaker combustion properties, and restricted volatility about diesel. Because biodiesel has a lower volatility and calorific value than diesel oil, this decline in BTE may be explained. At lower engine speeds, there is an increase in fuel consumption and heat loss. When compared to diesel fuel, methyl ester’s thermal efficiency decreases by 24% at 1500 rpm and maximum load.

### Air-fuel ratio

**Fig. 8:**
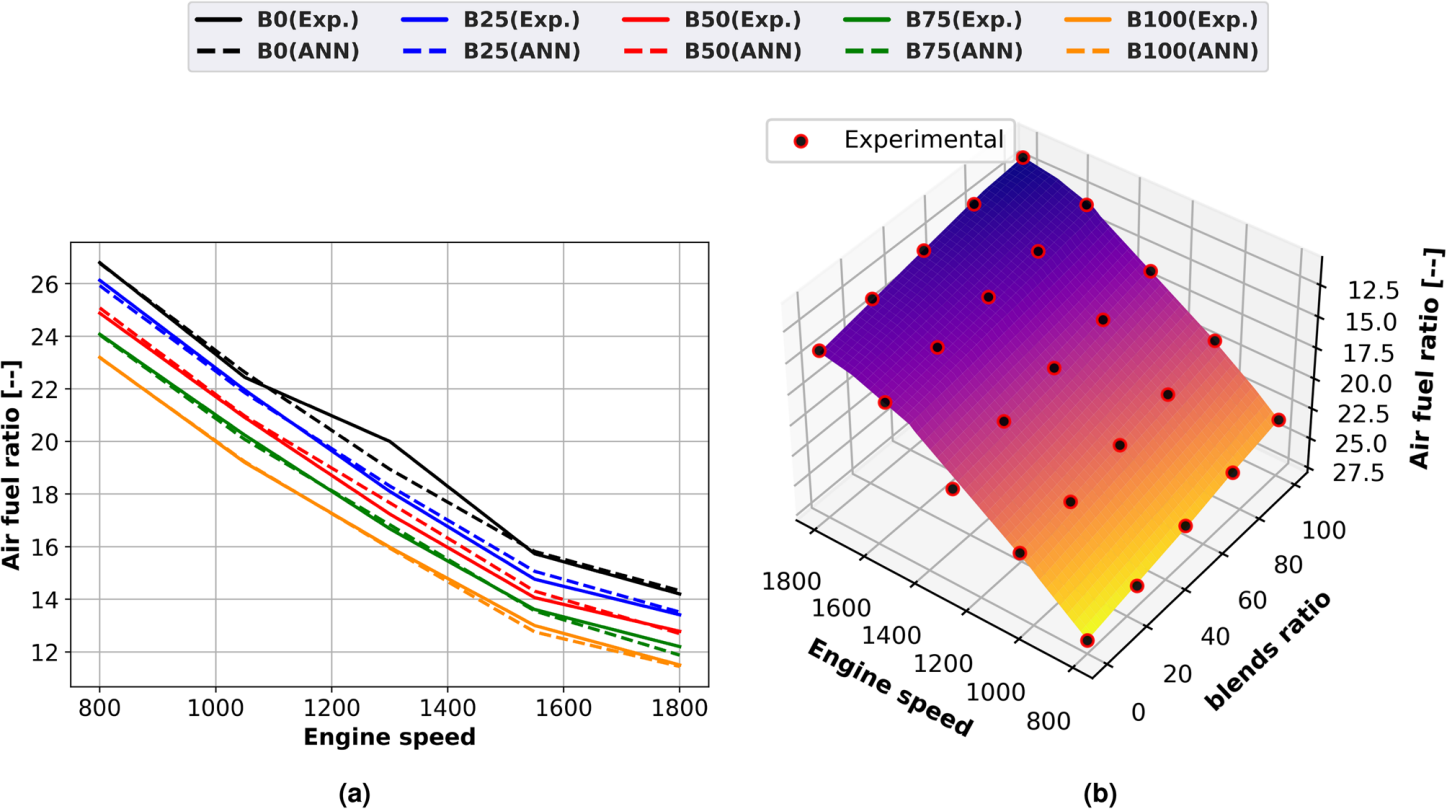
Air-fuel ratio of methyl ester mixtures at engine speed change (**a**) line plots for Experimental and ANN predicted performance, (**b**) experimental scatter and surface plots of the ANN model.

For diesel and biodiesel mixes, the fuel-air equivalency ratio changes with engine speed are predicted and shown as line and surface plots with experimental scatter in Fig. 8a and b, respectively. The equivalence ratio rises as engine speed increases. Adjusting the equivalence ratio is necessary because a greater engine speed results in a higher fuel flow rate. Due to diesel fuel’s higher calorific value and slower fuel consumption rate, biodiesel fuel has the lowest equivalence ratios. Diesel fuel has a higher stoichiometric air-fuel ratio than blends of biodiesel. Because biodiesel blends contain 11% more oxygen than diesel, they require less air to operate. The Biodiesel blend’s density increases with the increase of methyl ester ratio. Compared to diesel fuel, blends of biodiesel have a lower stoichiometric air-fuel ratio. Fuel consumption for a given volume increases and the true air-fuel ratio decreases when biodiesel is added. At full load and 1500 rpm engine speed, the methyl ester equivalency ratio is 15% lower than that of diesel oil.

### Volumetric efficiency

**Fig. 9:**
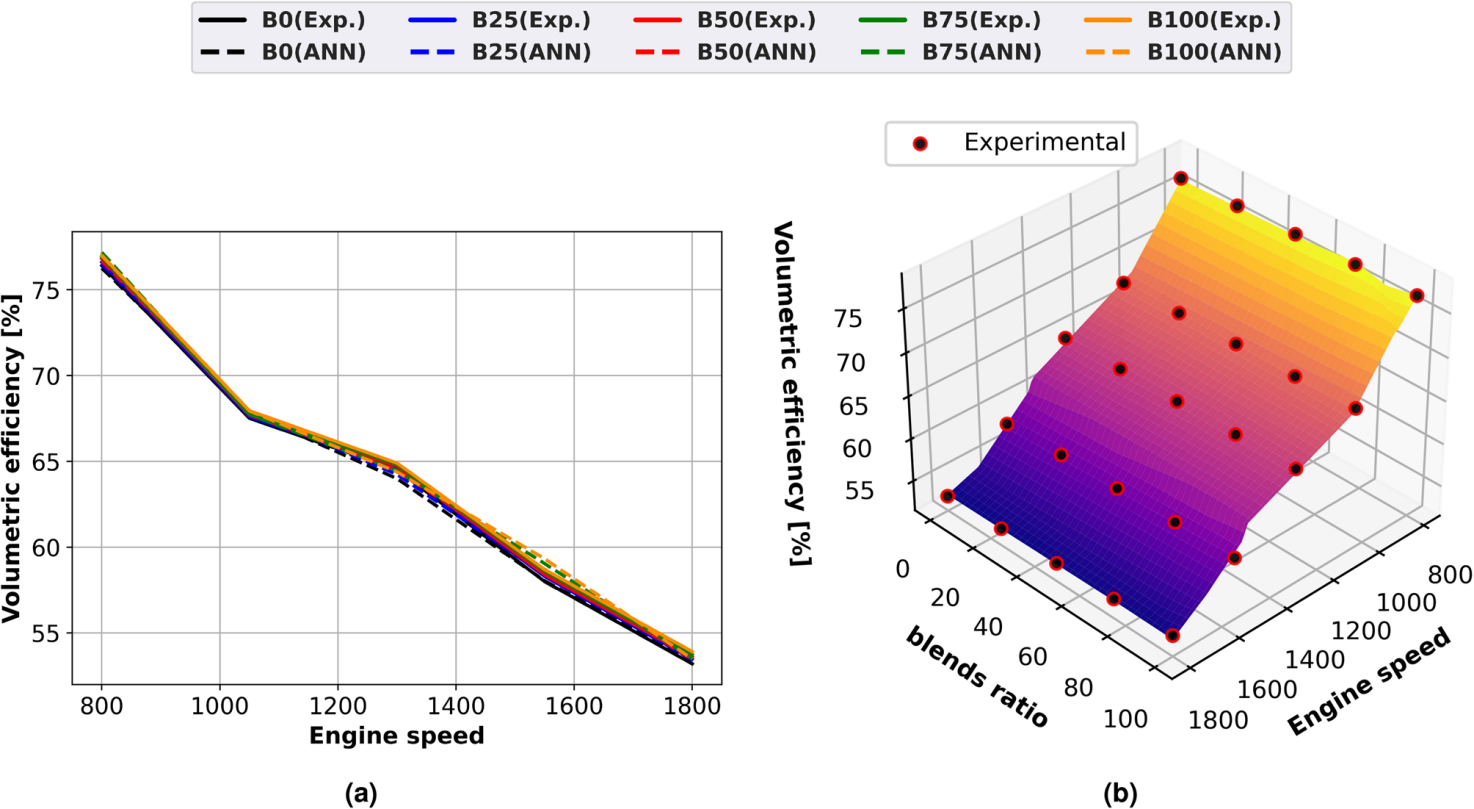
Volumetric efficiency of biodiesel mixtures at engine speed variation (**a**) line plots for Experimental and ANN predicted performance, (**b**) experimental scatter and surface plots of the ANN model.

Figure 9 illustrates the change in volumetric efficiency for both expected and actual performance when utilizing diesel oil and biodiesel mixtures as a function of engine speed using line graphs (Fig. 9a) and surface plots (Fig. 9b) with experimental scatter. Because of flow constraints in the air filter, intake manifold, and intake valves at higher engine speeds, it diminishes as engine speed increases. This causes less perfect air to enter the cylinder. Mixtures of biodiesel with more ethyl ester have lower volumetric efficiency. All methyl ester combinations have exhaust gas temperatures that are greater than those of diesel fuel. Because methyl ester fuel contains oxygen, it burns with less air. Compared to diesel fuel, the biodiesel blend uses less air due to the 11% weight percentage of oxygen in it. Engine speed has a major effect on volumetric efficiency due to the greater temperature of the residual gas. Due to severe air throttling at higher engine speeds brought on by limited airflow in the air filter, intake manifold, and intake valve, it gets smaller as engine speed increases. Due to variations in thermal characteristics and latent heat of vaporization, the biodiesel combination has lower input air temperature and greater cylinder temperatures than diesel fuel. B100’s volumetric efficiency was 4% less than diesel oil’s.

### CO emission

**Fig. 10:**
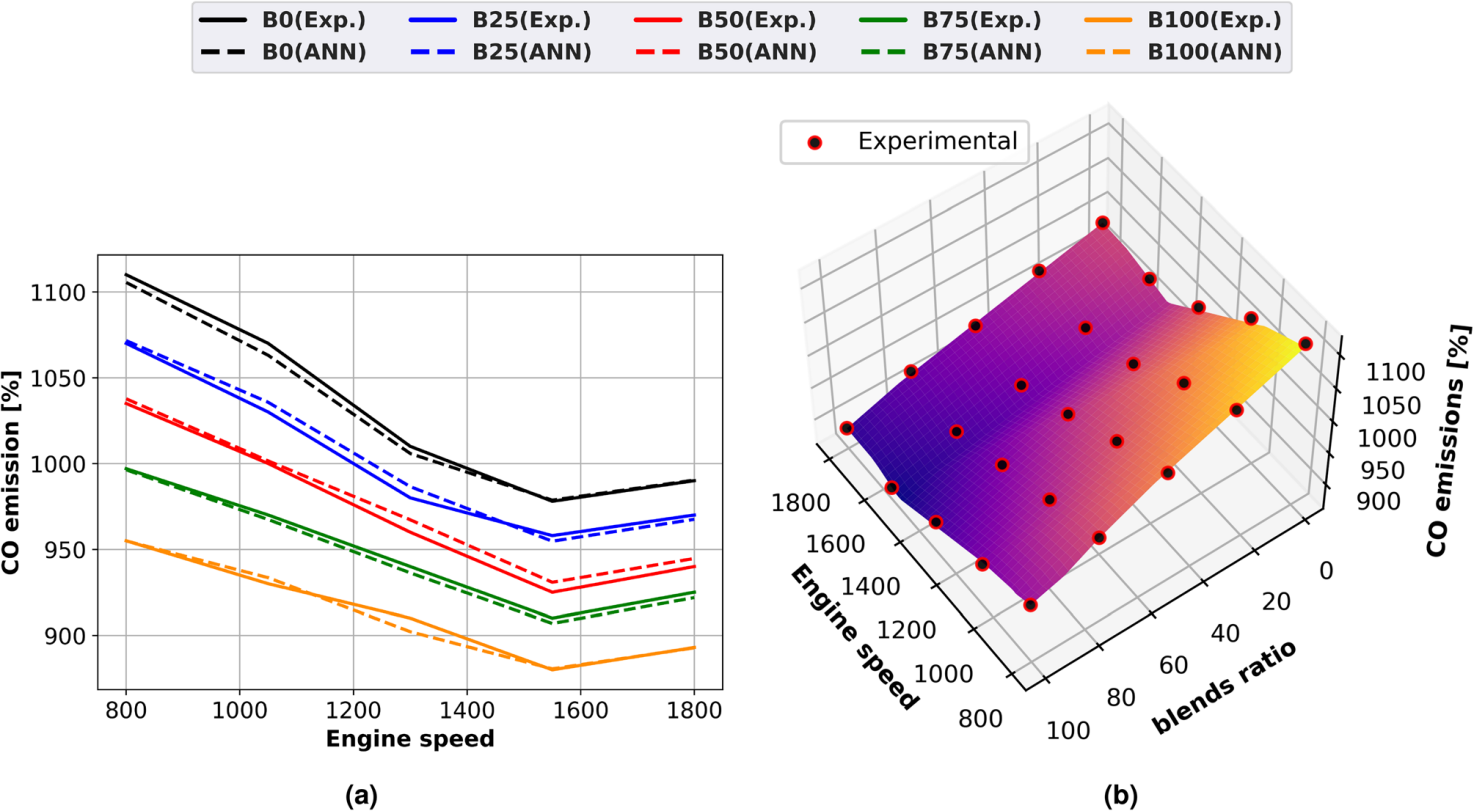
CO emission of biodiesel mixtures at different engine speeds (**a**) line plots for Experimental and ANN predicted performance, (**b**) experimental scatter and surface plots of the ANN model.

Blends of biodiesel and diesel Engine speed affect CO emissions, as shown in Fig. 10, which displays both actual and predicted results using line graphs in Fig. 10a and surface plots with experimental scatter in Fig. 10b. Engine speed increases cause CO emissions to fall until they reach a minimum and then begin to climb. Engine speed affects the amount of carbon monoxide produced because lower engine speeds promote slower rates of CO oxidation due to lower gas cylinder temperatures. Lower gas cylinder temperature and engine speed promote a slower rate of CO oxidation. Diesel fuel produces a higher proportion of CO than biodiesel. The reason for this is that the oxygen included in biodiesel molecules encourages combustion and reduces the likelihood that fuel-rich zones would emerge. Biodiesel has been discovered to have greater air-fuel mixing and combustion efficiency than diesel oil because it includes more oxygen. When compared to diesel fuel, B100 reduces CO emissions by 12% at maximum braking power and engine speed of 1500 rpm.

### HC emission

**Fig. 11:**
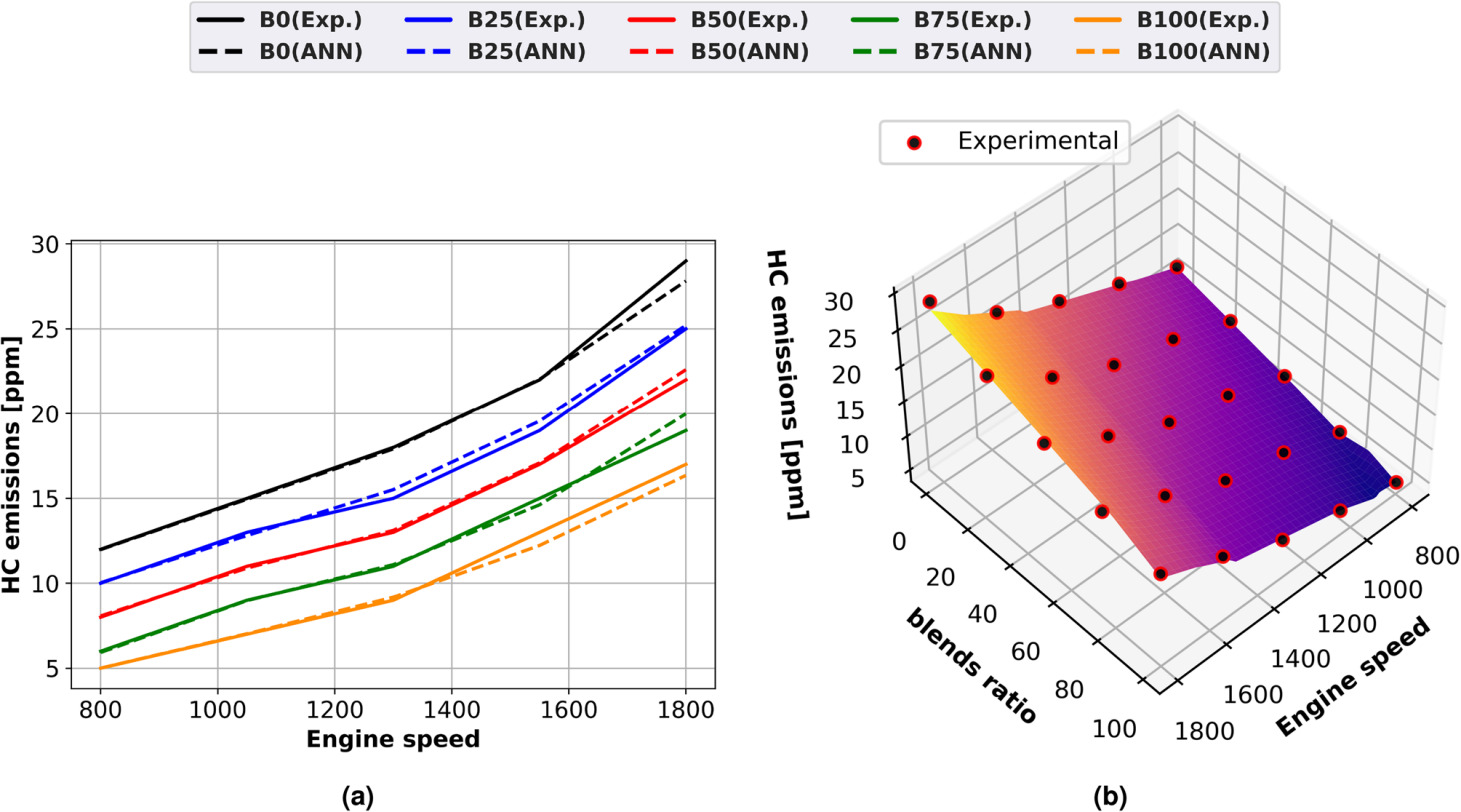
HC concentrations of biodiesel mixtures with engine speed variation (**a**) line plots for Experimental and ANN predicted performance, (**b**) experimental scatter and surface plots of the ANN model.

Figure 11 shows the relationship between HC emissions and engine speed for diesel and methyl ester for both experimental and ANN projected data using surface plots with experimental scatter (Fig. 11b) and line graphs (Fig. 11a). Higher engine speeds, more fuel consumption, and higher cylinder temperatures all result in increased HC emissions from all biodiesel blends. Reduced engine speeds produced lower HC emissions. The reason for this is the high engine load, which causes a rich fuel mixture and an oxygen shortage. Methyl ester lowers hydrocarbon emissions at all engine loads because of its high oxygen content. Biodiesel emits less hydrocarbons than diesel fuel because it has a higher cetane number and decreased ignition delay. When running at maximum load at 1500 rpm and using biodiesel instead of diesel fuel, HC emissions are reduced by 44%.

### NOx emission

**Fig. 12:**
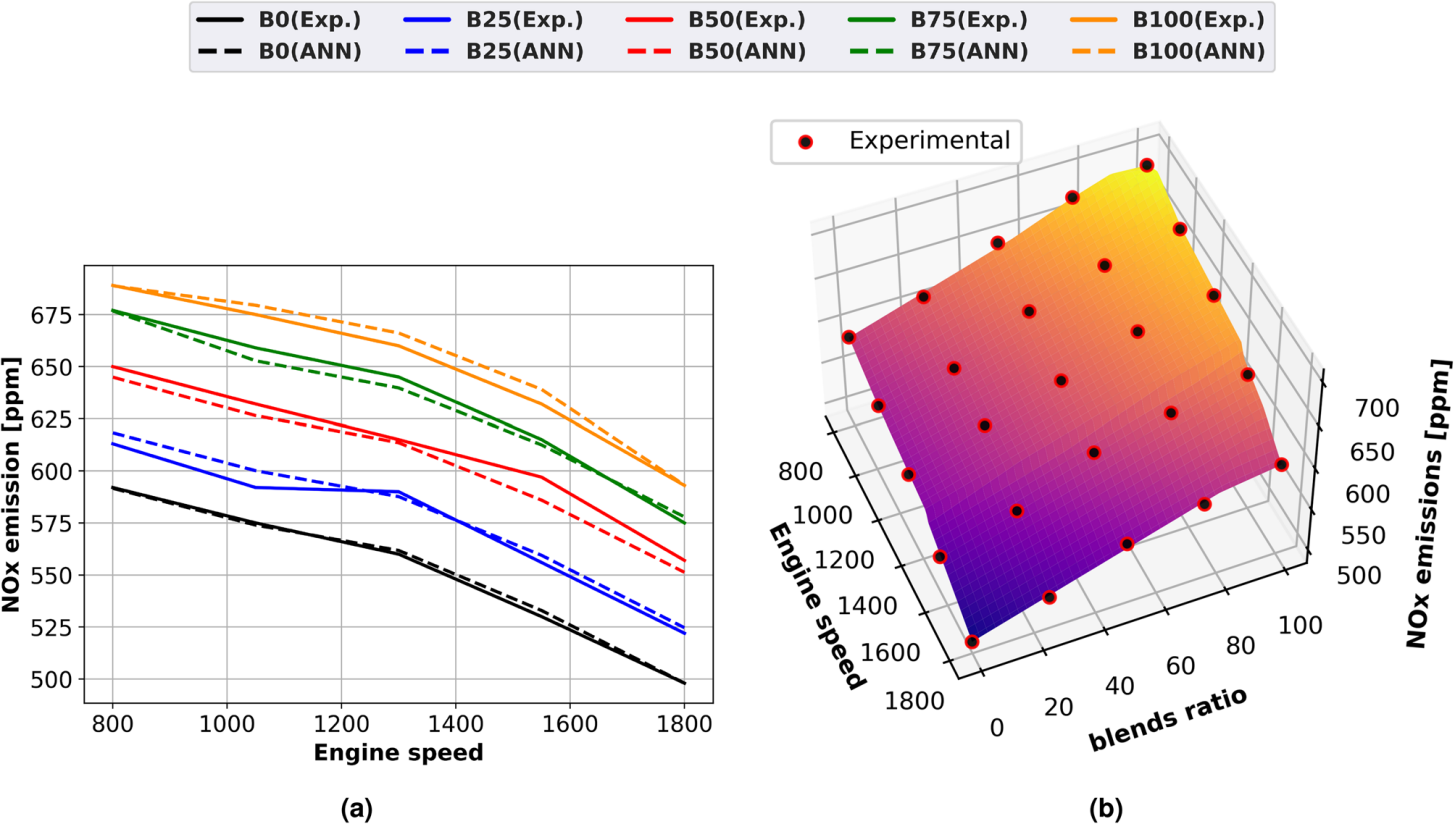
NOx emissions of methyl ester mixtures at different engine speeds (**a**) line plots for Experimental and ANN predicted performance, (**b**) experimental scatter and surface plots of the ANN model.

Figure 12 uses expected and experimental data to show how engine speed affects methyl esters mixes on NOx emissions using surface plots with experimental scatter (Fig. 12b) and line graphs (Fig. 12a). The production of thermal NOx is influenced by the cylinder’s oxygen level, temperature, and retention duration. The fuel-air mixture is lean when the engine is running smoothly and rich when it is running vigorously. A rise in cylinder temperature is a reason for the increase in NOx emissions. A richer fuel-air mixture was produced by the turbulence that the faster engine speed produced within the cylinder. Higher cylinder combustion temperatures cause the combination of dissociated nitrogen and oxygen to produce thermal NOx. As the methyl ester proportion rose, so did NOx emissions. The cylinder’s temperature and oxygen concentration caused thermal NOx to be produced. All of this causes the adiabatic flame’s temperature to rise dramatically, which raises the nitrogen oxide emissions from biodiesel in comparison to pure diesel. A richer A/F mixture is produced as the turbulence inside the engine cylinders increases. An increase in thermal NOx has been associated with reductions in air mixing, ignition delay, and fuel preparation time. At full load and 1500 rpm, B100 generates 23% more NOx than diesel oil.

### Smoke emission

**Fig. 13:**
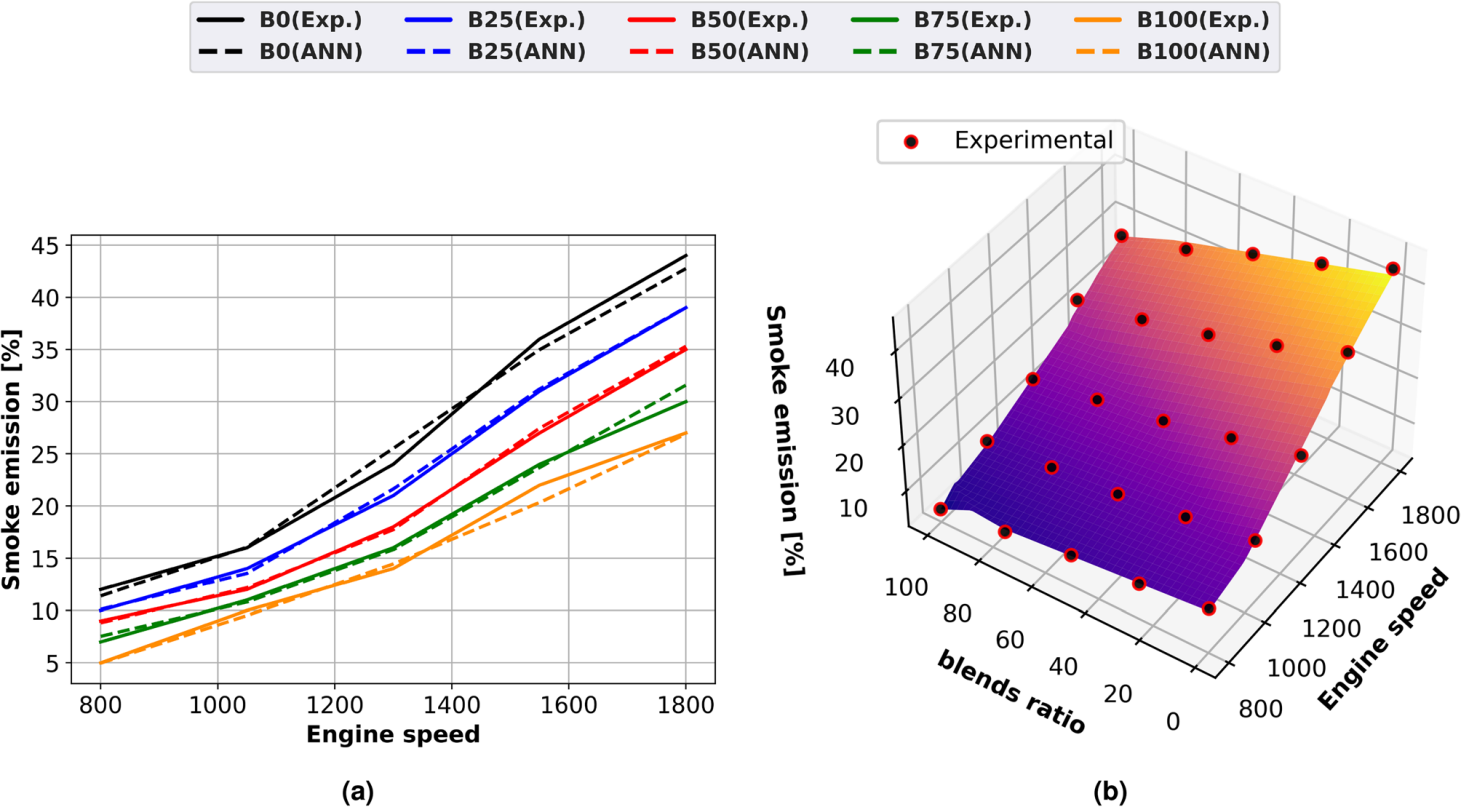
Smoke emission of biodiesel mixtures at different engine speeds (**a**) line plots for Experimental and ANN predicted performance, (**b**) experimental scatter and surface plots of the ANN model.

Line graphs (Fig. 13a) and surface plots (Fig. 13b) with experimental scatter are used in Fig. 13 to illustrate how engine speed and various biodiesel blends affect smoke emissions. Both experimental and predicted results are agreed upon. Because more diesel oil was being used, the engine’s speed increased and smoke emissions rose as a result. Reduced engine speeds result in less smoke production since there is more oxygen present. At high engine speeds, the oxygen content falls and observable smoke emissions happen as a result of the increased fuel consumption. As the amount of biodiesel grew, the amount of smoke created by methyl ester dropped. The lower smoke emission was caused by the biodiesel’s oxygen concentration. Smoke emissions rose with output power and fuel use. When methyl esters were added to ordinary diesel, the amount of smoke produced was decreased. Because biodiesel contains oxygen, combustion and smoke quality are enhanced. Biodiesel has higher particle oxidation and greater smoke-reducing capability during diffusion combustion since it burns more slowly due to the oxygen it contains. Oxygen is present in methyl esters, which enhances ignition, lowers smoke, and increases combustion efficiency. B100 has 48% reduction in smoke emissions linked to diesel oil when a diesel engine is running at 1500 rpm at full load.

### In-cylinder pressure

**Fig. 14:**
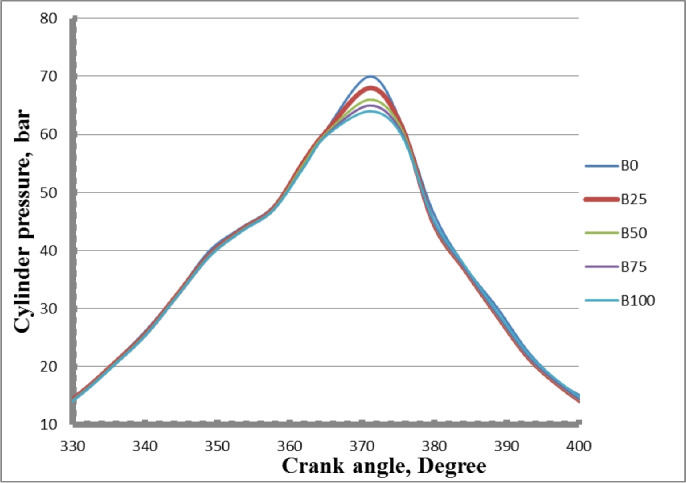
Cylinder pressure of biodiesel blends at different crank angles.

Figure 14 shows how a diesel engine’s internal pressure varies with crank angle while it is operating at maximum power, using both different mixtures of biodiesel fuel and pure diesel. All of the fuels that were examined showed comparable patterns in cylinder pressure. Blends of biodiesel have lower peak cylinder pressure than diesel. The cylinder pressure is lowered while using methyl ester oil since it ignites faster than regular diesel oil and has a higher cetane number. More biodiesel was held in the cylinder before the ignition delay decreased. The highest value of peak cylinder pressure for the diesel test fuel was achieved by extending the ignition delay period and collecting additional fuel. The oxygen content of biodiesel fuel decreases ignition delay and speeds up fuel droplet evaporation. Diesel oil burns more quickly and the fuel injection system sounds louder when using biodiesel because of its higher bulk modulus. Reduced calorific value in methyl ester blends containing diesel and inadequate atomization procedure might result from the cylinder pressure drop because it ignites more quickly than diesel fuel. Diesel used up less fuel during the diffusion phase than methyl ester did. The fact that biodiesel and its blends are denser and more viscous than diesel fuel, which significantly changes spray properties, exacerbates the atomization and vaporization problems. For B0, B25, B50, B75, and B100, the corresponding peak cylinder pressure values at full load were 70, 68, 66, 65, and 64 bars, respectively.

### Heat release rate

**Fig. 15:**
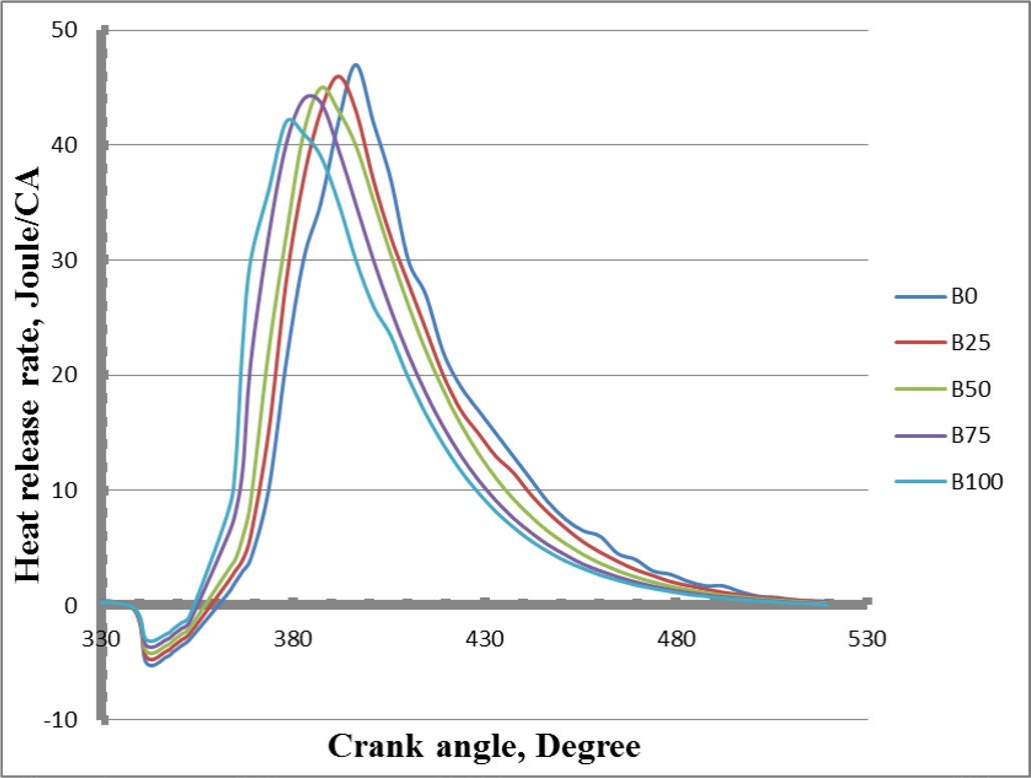
Heat release rate of biodiesel mixtures for tested fuels at different crank angles.

The heat release rate (HRR) for diesel and biodiesel fuel blends at various crank angles and full load is shown in Fig. 15. The patterns that were seen showed similarities between mixes of diesel and biodiesel. The peak heat release rates of mixed biodiesel were found to be lower than those of pure diesel when the methyl ester concentration was increased. As opposed to regular diesel fuel, biodiesel has a greater cetane value and shorter ignition delay. Since methyl ester blends require less fuel than pure diesel fuel, the greatest rate of heat release (HRR) in the premixed combustion phase is often lower. Because methyl ester combinations have a lower calorific value than crude diesel, they produce less heat. The heat release rate and cylinder pressure gradually decreased as the amount of biodiesel added to diesel fuel rose. Compared to diesel fuel, biodiesel is more viscous and dense. Biodiesel fuel uses less fuel in the first diffusion phase but more fuel in the succeeding premixed phase as compared to diesel oil. The short premixed combustion phase in biodiesel mixtures causes early burning. Blends of high-cetane biodiesel improve fuel preparation in the early phases before combustion, which causes combustion at the start of the compression stroke. There were problems with atomization, fuel evaporation, and inadequate fuel-air mixing because the methyl ester ignited more quickly. Because biodiesel fuel has a greater cetane number than diesel oil, methyl ester mixtures ignite faster. For B0, B25, B50, B75, and B100, the maximum HRRs were 47, 46, 45, 44, and 42 Joule/CA, respectively.

### Comparative study between experimental and developed model

**Fig. 16:**
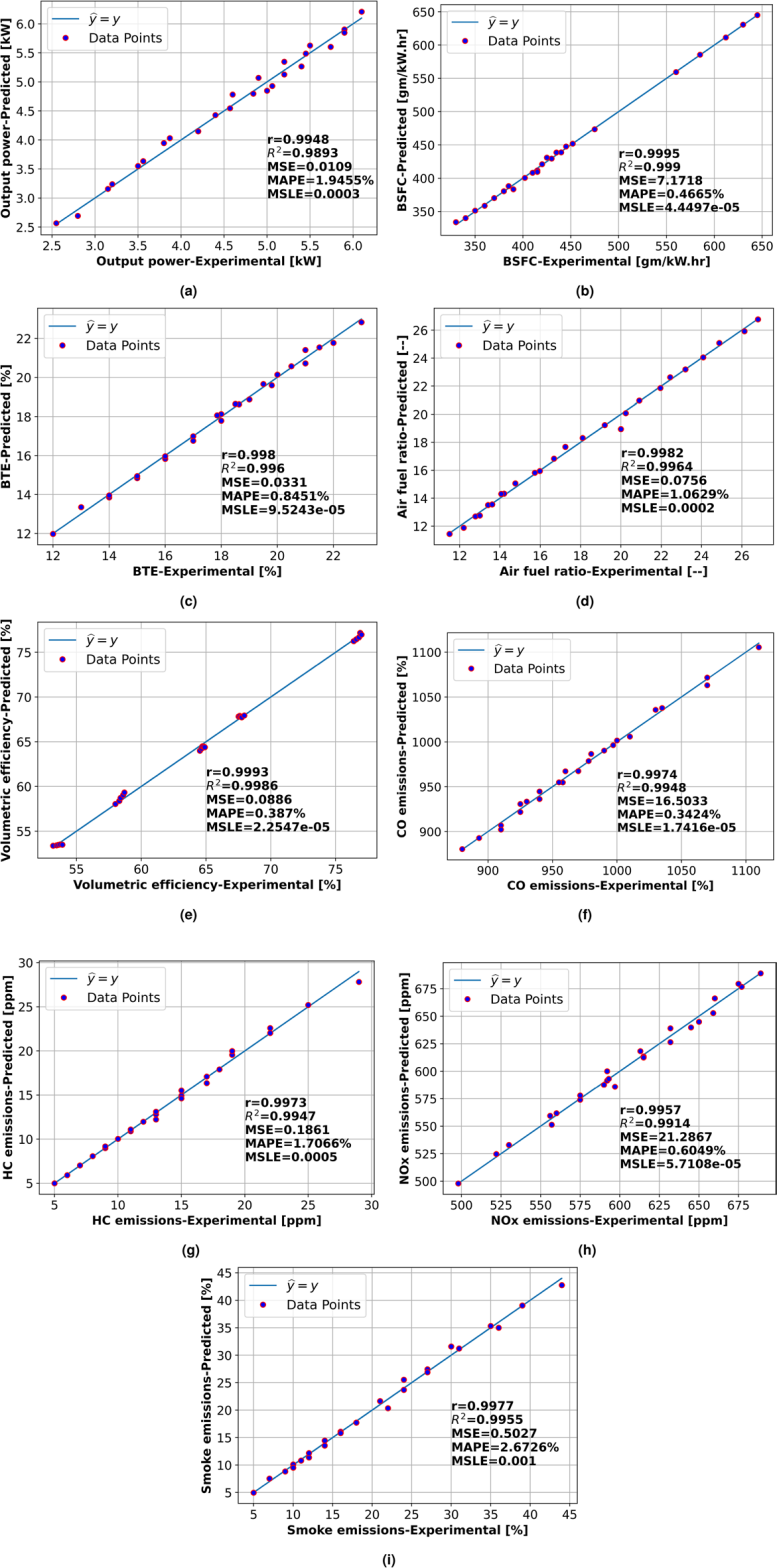
Regression analysis of engine performance and emission parameters (**a**) brake output power, (**b**) brake specific fuel consumption, (**c**) brake thermal efficiency, (**d**) air-fuel ratio, (**e**) volumetric efficiency, (**f**) CO emission, (**g**) HC emission, (**h**) NOx emission and (**i**) smoke emission.

The ability of the established *ANN* network to forecast engine performance and emission parameters is depicted in Fig. 16. Plotting was performed to compare the experimental data with the predicted data obtained from the ANN network. The exceptional network performance is indicated by the toeing of the data points on the $\widehat{y}=y$ line (line slope equal to $45^{\circ }$). The Regression analysis of engine performance and emission parameters is shown in Fig. 16 were examined using equations 7-15. Fig. 16 showed that the experimental and ANN predicted values are extremely close together, demonstrating the model’s excellent efficiency. Extremely higher values of Correlation coefficient (*r*) of 0.9948, 0.9995, 0.998, 0.9982, 0.9993, 0.9974, 0.9973, 0.9957 and 0.9977 were observed for brake output power (Fig. 16a), brake specific fuel consumption (Fig. 16b), brake thermal efficiency (Fig. 16c), air-fuel ratio (Fig. 16d), volumetric efficiency (Fig. 16e), CO emission (Fig. 16f), HC emission (Fig. 16g), NOx emission (Fig. 16h) and smoke emission (Fig. 16i) respectively. Also, higher values of coefficient of determination $R^2$ of (0.9893, 0.999, 0.996, 0.9964, 0.9986, 0.9948, 0.9947, 0.9914, and 0.9955 respectively) were achieved. Extensively low values of Mean squared error (*MSE* = 0.0109, 7.1718, 0.0331, 0.0756, 0.0886, 16.5033, 0.1861, 21.2867, and 0.5027 respectively) were obtained. Also mean absolute percentage errors *MAPE* were lower than 3$\%$ for all output variables (*MAPE* = 1.9455$\%$, 0.4665$\%$, 0.8451$\%$, 1.0629$\%$, 0.387$\%$, 0.3424$\%$, 1.7066$\%$, 0.6049$\%$ and 2.6726$\%$, respectively). Remarkably low values of mean squared logarithmic error *MSLE* lower than 0.002 were achieved (*MSLE* = 0.0003, 4.4497E−05, 9.5243E−05, 0.0002, 2.2547E−05, 1.7416E−05, 0.0005, 5.7108E−05 and 0.001, respectively).

**Fig. 17:**
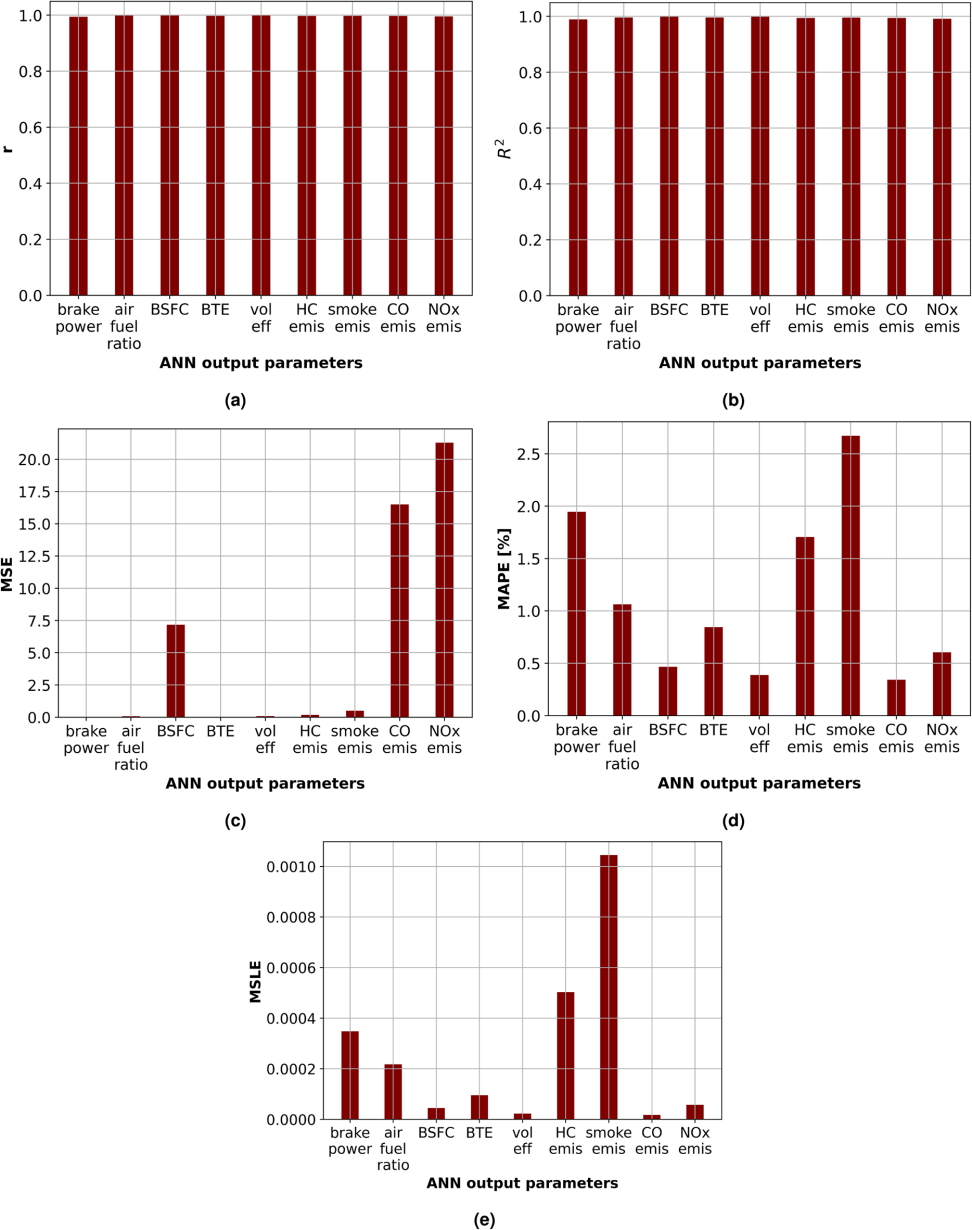
ANN predictive performance metrics: (**a**) r, (**b**) $R^2$, (**c**) MSE, (**d**) MAPE and (**e**) MSLE.

Figure 17 provides a summary of the ANN predictive performance metrics which consists of correlation coefficient (*r*), coefficient of determination ($R^2$), mean squared error (*MSE*), mean absolute percentage error (*MAPE*) and mean squared logarithmic error (*MSLE*) for ANN output parameters which represent engine performance and emission characteristics: brake output power, air-fuel ratio, brake specific fuel consumption(BSFC), brake thermal efficiency (BTE), volumetric efficiency, HC emission, smoke emission, CO emission and NOx emission. Better ANN model prediction performance is indicated by higher scores of *r* and $R^2$ as well as lower *MSE*, *MAPE*, and *MSLE* values. The ANN model showed outstanding performance for all output parameters. All values of correlation coefficients (*r*) were greater than 0.99 as shown in Fig. 17a, proving a highly robust linear relation between the experimental and estimated values. Furthermore, Fig. 17b shows that the scores of $R^2$ demonstrated a level above 0.98 for all engine output parameters which suggests that over 98$\%$ of the variability in the *ANN* output parameters can be determined by the model. The mean squared error (*MSE*) illustrated in Fig. 17c showed minimum values for brake output power(*MSE* = 0.0109), brake thermal efficiency (*MSE* = 0.0331), air-fuel ratio (*MSE* = 0.0756), and volumetric efficiency (*MSE* = 0.0886) denoting minimal squared deviations between the experimental and *ANN* estimated values for these parameters, while it gives relatively highest values for NOx emission and CO emission (*MSE* = 21.2867 and 16.5033 for NOx and CO emission respectively). The error’s magnitude in percentage forms is represented by the mean absolute percentage error (*MAPE*) as shown in Fig. 17d. The minimum value for CO emission (*MAPE* = 0.3424$\%$) and maximum value for Smoke emission (*MAPE* = 2.6726$\%$) were shown. This metric signifies the *ANN* performance prediction was best for CO emission and needs some enhancement to predict smoke emission. The mean squared logarithmic error (*MSLE*) shown in Fig. 17e followed a similar trend as *MAPE*, with the minimum value for CO emission (*MSLE* = 1.7416e−05) and the highest for smoke emission (*MSLE* = 0.001). This metric signifies the deviation between the experimental and predicted log-transformed values. The results illustrate the efficiency of the ANN model for accurate prediction of the engine parameters. Higher *r* and $R^2$ scores for all parameters imply a noteworthy model predictive performance. However, various ranges of *MSE*, *MAPE*, and *MSLE* values indicate that the model performs differently for various output parameters. Although the model demonstrated remarkable performance for CO emission, NOx emission, and volumetric efficiency predictions, it was slightly less accurate for smoke emission.

Discrepancies between Artificial Neural Network (ANN) predictions of diesel engine performance and emissions and experimental results often arise due to several factors. Firstly, the ANN model may not capture the full complexity of the engine’s physical and chemical processes, leading to inaccuracies in prediction. This is often due to insufficient or biased training data that fails to represent all operational conditions adequately. Secondly, the model might overfit the training data, performing well on known data but poorly on new, unseen data. Thirdly, inaccuracies in sensor measurements and data recording during experiments can introduce noise that the ANN struggles to generalize. Lastly, simplifications and assumptions in the ANN architecture, such as the choice of network design, might not fully align with the intricate dynamics of engine performance and emissions. Addressing these discrepancies involves enhancing data quality, improving model complexity and architecture, and ensuring better representation of real-world conditions in the training dataset.

Discrepancies in the performance of Artificial Neural Networks (ANNs) when predicting different output parameters for diesel engine performance and emissions can stem from several factors. Firstly, the complexity and non-linearity of the relationships between input variables and output parameters vary; some outputs may have more straightforward dependencies, while others involve intricate interactions. Secondly, the quality of training data can influence ANN performance, with some parameters potentially having more comprehensive and higher-quality datasets. Thirdly, the inherent variability and noise in the data associated with certain outputs, such as emissions, which can be influenced by numerous external factors, may affect predictive accuracy. Additionally, the architecture of the ANN, including the number of layers and neurons, might be more suitable for certain outputs over others. Lastly, the feature engineering process, which involves selecting and transforming input variables, can also impact the model’s ability to accurately predict different parameters, as some features may be more relevant to specific outputs.

Even with small discrepancies, the ANN is a powerful prediction tool. The results motivate additional research into this model and offer possible directions for enhancing its accuracy for various output parameters. To improve performance even more, future research could investigate different neural network designs or data preprocessing methods.

## Conclusions

Waste cooking oil (WCO) is utilized directly in diesel engines in this investigation, and the combination of biodiesel and diesel exhibits unique properties according to ASTM. Diesel and biodiesel can be mixed in the following ratios: 25$\%$, 50$\%$, 75$\%$, and 100$\%$. In an experimental investigation comparing engine characteristics and emissions for mixtures of diesel and biodiesel, *ANN* mathematical model was used to forecast engine performance and exhaust emissions for various combinations of engine speed and fuel mixtures given a collection of experimental input-output data. The following might be used to summarize the outcomes:

- The B100’s output power is 25$\%$ less than the diesel engine’s power at full load and 1500 rpm. Biodiesel fuel yielded the highest gains in BSFC at 1500 rpm, at 23$\%$, when compared to diesel fuel. Methyl ester’s thermal efficiency decreases by 24$\%$ when compared to diesel fuel at 1500 rpm and maximum load. When compared to diesel, biodiesel performs 4$\%$ less efficiently in terms of volumetric efficiency and 15$\%$ in terms of air-fuel ratio at 1500 rpm.
- At maximum braking power and engine speed of 1500 rpm, B100 decreases CO, HC, and smoke emissions by 12$\%$, 44$\%$, and 48$\%$, respectively, in comparison to diesel fuel. When running at 1500 rpm with full load, B100 produces 23$\%$ higher NOx than diesel fuel.
- For B0, B25, B50, B75, and B100, the corresponding peak cylinder pressure values at full load were 70, 68, 66, 65, and 64 bar, respectively but the maximum HRR were 47, 46, 45, 44, and 42 Joule/CA, respectively.
- The ANN model showed outstanding predicting performance for all engine output parameters. All values of correlation coefficients (*r*) were higher than 0.99, the $R^2$ scores showed a level over 0.98 for all parameters accompanied by extremely lower values of *MSE*, *MAPE*, and *MSLE* imply that the ANN model has a considerable predictive performance.
- The mean squared error (*MSE*) showed minimal values for brake output power, volumetric efficiency, air-fuel ratio, and brake thermal efficiency, while it gives maximal values for CO and NOx emissions. Mean absolute percentage error (*MAPE*) and mean squared logarithmic error (*MSLE*) were smallest for CO emission and highest for smoke emission.
- High density and viscosity of pure biodiesel compared to diesel oil leads to atomization and vaporization problems. These problems produce the fuel injector coking and filter plugging in cold climates. The lower calorific value of biodiesel than diesel oil leads to lower output power, higher fuel consumption, and thermal efficiency loss. The higher flash point of biodiesel than pure diesel achieves safe storage and handling. Oxygen content in biodiesel improves combustion efficiency and leads to reductions in CO and HC emissions. NOx emission of biodiesel is higher than diesel oil and can be reduced by exhaust gas recirculation. Waste cooking biodiesel of 20% is recommended as an alternative fuel in diesel engines because of its near chemical and physical properties to diesel oil.

## Data Availability

All data generated or analyzed during this study are included in this published article.

## Abbreviations

${\bar{y}}$: Average (mean) value of the data
$\bar{{\widehat{y}}}$: Average (mean) value of the predicted values of the data points
$$\Phi$$: Activation function
$\widehat{{y}_{i}}$: Predicted value of the data
$\zeta$: Number of ANN layers
*ANN*: Artificial neural network
*b*: Bias
*BSFC*: Brake specific fuel consumption
*BTE*: Brake thermal efficiency
*CO*: Carbon monoxide,$\%$
*HC*: Hydrocarbons, ppm
*I*: Number of influencing parameters
*MAPE*: Mean absolute percentage error
*MSE*: Mean squared error
*MSLE*: Mean Squared Logarithmic Error
*N*: Number of data points
*Nox*: Nitrogen oxides, ppm
*r*: Correlation coefficient
*S*: Linear sum of the signal values
*SSR*: Sum of squared regression
*SST*: Sum of squares total
${n}^{\zeta }$: Number of neurons at $\zeta ^{th}$ layer
${R}^{2}$: Coefficient of determination
${w}_{i}$: Weight coefficients, or signal value
$${x}_{i}$$: Input parameter
${X}_{norm}$: Normalized input parameter
${y}_{i}$: Actual value of the data point
